# Susceptibility Rates of *Pseudomonas* Aeruginosa Strains to Quinolones

**DOI:** 10.4103/0974-2727.72217

**Published:** 2010

**Authors:** Luna Adhikari, K Roy, Dechen C Tsering, Ranabir Pal, Sumit Kar

**Affiliations:** Department of Microbiology, Sikkim Manipal Institute of Medical Sciences (SMIMS) and Central Referral Hospital, (CRH), 5^th^ Mile, Tadong, Gangtok, Sikkim, India; 1 Department of Community Medicine, Sikkim Manipal Institute of Medical Sciences (SMIMS) and Central Referral Hospital, (CRH), 5^th^ Mile, Tadong, Gangtok, Sikkim, India

Sir,

*Pseudomonas aeruginosa* is an important bacterial pathogen, particularly as a cause of infections in hospitalized patients and revealed trends of increasing antimicrobial resistance. However, resistance to fluoroquinolone antibiotics has been reported in recent years as well. This cross-sectional study included a total of 932 clinical samples received from various departments of Central Referral Hospital, Gangtok from August 2005 to August 2008. The samples were cultured in 5% sheep blood agar and MacConkey agar at 37°C for 18–24 hours. The isolates were preliminarily identified as *Pseudomonas* spp. by biochemical and physiological tests, colony morphology, production of diffusible pigments, and a grape-like odour. Gram-negative bacilli with above-mentioned characteristic features were considered as *P aeruginosa* on the basis of a positive oxidase test, a triple sugar iron (TSI) agar reaction of alkaline over no change, growth at 42°C and production of bright-blue to blue green diffusible pigment on Mueller-Hinton agar. Non-pigmented isolates were identified as *P. aeruginosa* by the above-mentioned characters along with oxidation of glucose, but not disaccharides, hydrolysis of acetamide, and reduction of nitrates to nitrogen gas. One sample was collected from each patient. The susceptibility of *P aeruginosa* strains to ciprofloxacin (5 µg), ofloxacin (5 µg), levofloxacin (5 µg), norfloxacin (10 µg), and pefloxacin (5 µg) was studied by Kirby-Bauer disc diffusion method according to NCCLS (National Committee for Clinical and Laboratory Standards) criteria. Out of a total of 932 clinical samples received, 682 samples were pus, 125 were urine, 83 were blood, and 42 were sputum. Of them 268 (28.76%) were isolated having *P aeruginosa* strains [pus (196), urine (36), blood (24), and sputum (12)]. Two hundred and eight (77.61%) isolates were obtained from in-patients, while only 60 (22.39%) were from out-patients. Ciprofloxacin (47.76%) was found to be the most effective anti-pseudomonal agent followed by levofloxacin (44.78%). However, this difference was not significant (*P*>0.05). The susceptibility patterns of ciprofloxacin and levofloxacin were highly significant when analyzed with the susceptibility patterns of norfloxacin (23.88%), ofloxacin (20.90%), and pefloxacin (8.96%), (Chi-square: 225.75, *p* value <0.0001). Belgian study reported that the resistance rate of P aeruginosa to ciprofloxacin had gradually increased over years (13% in 1991, 16–18% in 1993–1995, 30% in 1998–1999).[1] Other studies from India reported ciprofloxacin resistance rate to be between 12.5% and 30.7%.[2,3] Levofloxacin resistance has been reported to be between 16.9% and 36% in different studies.[2,4] Ofloxacin resistance has been reported by other researchers to be between 19% and 62.5%.[2,5]

Since fluoroquinolones are used as a monotherapeutic agent in non-intensive care unit patients in our hospital, the finding of the research indicated that improved antibiotic stewardship and infection control measures will be needed to prevent or slow the emergence and spread of multidrug-resistant, non-fermenting gram-negative bacilli in the resource-poor healthcare setting. Selecting appropriate antibiotics and optimizing their use on the basis of pharmacodynamic concepts remains the best way of coping with pseudomonal infections.
